# Impact of nano-morphology, lattice defects and conductivity on the performance of graphene based electrochemical biosensors

**DOI:** 10.1186/s12951-019-0535-6

**Published:** 2019-10-03

**Authors:** Teddy Tite, Elena Alina Chiticaru, Jorge S. Burns, Mariana Ioniţă

**Affiliations:** 1 Faculty of Medical Engineering, University Politehnica of Bucharest, Gh Polizu 1-7, 011061 Bucharest, Romania; 2 Advanced Polymer Materials Group, University Politehnica of Bucharest, Gh Polizu 1-7, 011061 Bucharest, Romania

**Keywords:** Graphene, Electrochemistry, Biosensors, Morphology, Lattice defects

## Abstract

Diverse properties of graphenic materials have been extensively explored to determine properties that make good electrochemical nanomaterial-based biosensors. These are reviewed by critically examining the influence of graphene nano-morphology, lattice defects and conductivity. Stability, reproducibility and fabrication are discussed together with sensitivity and selectivity. We provide an outlook on future directions for building efficient electrochemical biosensors.

## Highlights

- Fabrication of electrochemical biosensors
- Influence of nano-morphology, lattice defects and conductivity
- Concepts and parameters governing electrochemical biosensor sensitivity, selectivity, stability and reproducibility

## Introduction

Biosensors, broadly applicable to biology and biomedicine, can transform healthcare with innovative precise detection of scarce analytes via a biorecognition element and a transducer [1, 2]. Specific detection between an analyte (e.g. protein, enzyme, nucleic acid, biomarker molecule) and corresponding aptamer (apt) bioelement is transformed into a measurable signal by a transducer defining the biosensor type (e.g. electrical, electrochemical, optical, thermal, piezoelectrical or magnetic) [3]. A desirable biosensor characteristic is real-time quantitative analyte concentration measurement within a complex environment [4]. Since Leland Clark’s 1962 vanguard biosensor, designs have advanced considerably. Advantages sought include high sensitivity and selectivity, low-cost, simplicity, rapid response, low background noise with a strong signal-to-noise ratio (S/N), allowing label-free and environmentally friendly techniques [5, 6]. Nanotechnology has introduced many advanced materials, such as quantum dots (QDs), carbon nanotubes (CNT), and lately two-dimensional materials such as graphene. With peak surface-to-volume ratios, 2D materials introduce interfacial phenomena that can greatly improve biosensor sensitivity, selectivity, response time, and limits of detection [7, 8]. Graphene may be considered a biosensor material of choice [9] and a benchmark for future electrochemical sensor nanomaterials, such as boron nitride, transition metal dichalcogenides (TMDs), black phosphorus (BP), silicene and antimonene [6, 7]. The extensive scientific exploration of graphene and TMDs has been comprehensively reviewed [10]. Lately, composite nanomaterials of more complex architecture have sought to improve electron transfer qualities and analyte sensitivity, however, their fabrication may differ between laboratories and subtle alterations in characteristics may confusingly influence properties and performance.

Here, we introduce concepts for the main techniques used in electrochemical biosensing, highlighting the distinguishing qualities of advanced nanomaterials used for electrochemical biosensor elaboration. For graphenic sensors in particular, we focus on three key parameters: morphology, electrical conductivity and the influence of lattice defects.

## Techniques used in electrochemistry

Electrochemical sensors represent promising and robust analytical tools, increasingly used in many fields, including analytical chemistry, molecular biology, healthcare diagnostics, environmental monitoring and security [2]. Electrochemical sensors generate electrical signals proportional to an analyte concentration. Conventionally, electrochemical events are often measured using a cell with three electrodes connected to a potentiostat: a working electrode (WE), a reference electrode (RE) and an auxiliary counter electrode (CE). Electrochemical processes involve charge transfer across the interface of the WE and an electrolyte. The three-electrode configuration allows measurement of a potential (current) between RE (CE) and WE with minimal ohmic drop interference. The current flowing through the RE is minimised to avoid its polarisation, thus stabilising the potential between the WE and RE.

Different electrochemical sensing technique nomenclature reflects what is measured: (1) Amperometric, current at a fixed potential; Voltammetric, current at a varying potential. (2) Potentiometric, potential or charge accumulation at constant current. (3) Conductometric, WE conductivity at varying frequencies. (4) Impedimetric, impedance (both resistance and reactance) at varying AC (alternating current) potential frequencies. (5) Field-effect, current generated by a potentiometric effect at the gate electrode using a transistor [11]. Typical voltammetry methods used in electrochemical biosensing include cyclic voltammetry (CV) [12], differential pulse voltammetry (DPV), and square wave voltammetry (SWV). Although electrochemical techniques are versatile, amperometry, voltammetry and electrochemical impedance spectroscopy (EIS) are favored in microfluidics and biosensing application [13].

In amperometry, the current (I) is recorded as a function of time (t) and various amperometric based sensors have been designed to measure the current continuouly from the oxidation or reduction of an electroactive species in an electrochemical reaction. The defining Clark experiment introduced the simplest form of amperometric biosensor, whereby currents produced by consumption of oxygen or production of hydrogen peroxide by oxydoreduction activities of glucose oxidase (GOx) permitted measurement of glucose concentration. Nowadays, amperometric biosensors are commercially available and widely used for glucose monitoring, reflecting the pioneering design’s effectiveness. Exemplifying current design progress, a miniaturised screen printed biosensor (100–400 microns), using a water-based ink containing cobalt phthalocyanine, detected glucose by chronoamperometry (CA) in the linear range from 0.5 to 2.5 mM [14], appropriate for cell toxicity applications.

Voltammetry, a leading electrochemistry technique, contrasts amperometry by being able to probe the reversibility of the studied system, since the electrode potential (E) changes as a function of time. Notably, [Fe(CN)_6_]^3−/4−^ is a standard electrolyte for electrochemical biosensing, because of higher surface sensitivity in comparison to other redox systems such as [Ru(NH_3_)_6_]^3+/2+^ [15]. In cyclic voltammetry, once the setting potential is reached, the working electrode’s potential is inversely ramped to return to the initial potential. Compared to other electrochemical methods, CV simply and quickly evaluates the working electrode efficiency, both qualitatively and moreover quantitatively via several useful parameters: (i) Surface area, using Randles–Sevcik equation; (ii) Reversibility of the electrochemical process, estimating the redox peak-to-peak potential separation difference (ΔE) (ΔE should be minimised, for one-electron process ΔE is 56.9 mV in theory); (iii) Heterogenous electron transfer rate constant (k° in cm s^−1^) using Nicholson equations [16, 17]; and (iv) Electrode transfer rate (K_s_ in s^−1^) from the Laviron equation [18]. Those parameters crucially determine the possible electron transfer speed and how it could influence biosensor sensitivity. Independent of the scan rate and electrode surface, the shape and current intensity of the CV curve change with analyte concentration. In DPV, the current is determined directly before each potential change (pulse period) and the current difference is represented as a function of the potential. DPV usefully eliminates the contribution of non-Faradaic (capacitive) processes, enhancing the precision of electrode reaction analysis, whilst minimizing the charging current for high sensitivity.

Various sensors based on CV and DPV transducer signal have been described [19]. Recently, CV and DPV were used to detect estriol (ET), one of four ovarian estrogens strongly influencing sexual and reproductive function. For CV curves, peak current increased linearly from 2 × 10^−6^ to 1 × 10^−4^ M ET and a detection limit of 8.7 × 10^−7^ M was achieved [20]. Innovatively, DPV was used to sense salbutamol sulfate (SBS), a bronchodilator for asthma treatment banned by anti-doping agencies due to ergogenic action and side-effects including tachycardia and arrhythmia at high doses. The peak current response to SBS concentration covered a 0.2 to 8 µM linear range with a detection limit of 6.8 × 10^−8^ M [21]. Chlorpromazine, used to treat depression, bipolar disorder and schizophrenia, can have serious overdose side-effects, including interpalpebral conjunctiva and cataracts. A suitable DPV sensor could monitor chlorpromazine doses in the range of 0.01 to 0.08 µM with a detection limit of 0.003 µM [22].

Electrochemical impedance spectroscopy, sometimes called AC impedance or impedance spectroscopy is a non-destructive and powerful technique providing time dependent quantitative data regarding the electrode processes and complex interfaces. Its growing popularity for biomolecule detection reflects a high interface binding event sensitivity. In EIS, both a DC (direct current) potential and a small sinusoidal AC perturbation potential (E_AC_ ~ 5–10 mV) are applied between the WE and the RE. The sinusoidal perturbations of the potential E(t) produces a sinusoidal current I(t) of the same frequency (ω) but shifted with a phase ϕ with respect to the potential. The magnitude |Z| and the phase angle ϕ of the recorded complex impedance reflects a function of the AC frequency after calculation using Ohm’s law (Z = V/I = |Z|e^jϕ^ = Z_real_ + jZ_imag_). The real part Z_real_ is similar to a resistance R (ϕ = 0, independent of frequency) while the imaginary part (Z_imag_) is the reactance (ϕ ≠ 0, dependent of frequency). In electrochemical biosensing, EIS is usually presented as a Nyquist plot, i.e. the dependence of Z_imag_ as a function of Z_real_. The diameter of the semi-circle in the middle frequency region of a Nyquist plot, representing charge transfer resistance (R_ct_), is related to the analyte concentration. Since the reactance is generally capacitive, i.e. of negative value,—Z_imag_ is always plotted for convenience. The dependence of |Z| and ϕ as function of AC frequency, respectively known as Bode amplitude and Bode phase plots are rarely described, yet they can provide additional relevant information. For quantitative analysis, Nyquist and Bode diagrams are usually modeled using Randles circuit [23]. Charge transfer resistance responsiveness to analyte concentrations yielded sensitive impedance biosensors [24]. For example, the impedimetric label-free aptamer biosensor for lysozyme detection showed a limit of detection (LOD) of 1.67 µM, a linear response up to 5 µM, and a sensitivity of 0.090 µM^−1^ in relative charge transfer resistance values [25]. Electrochemical transducers provide an attractive means of converting a biological event to an electric signal. Recently, more advanced biosensors have been fabricated coupling electrochemical techniques and nanotechnology.

## Toward the use of graphenic nanomaterials in biosensing

### Standard carbonaceous electrodes

Numerous reviews highlight use of carbon versus mercury, platinum or gold for advanced electrochemical electrodes, since carbon materials are relatively non-toxic, chemically inert, cost-effective, having a wide potential window and provide good stability. Highly biocompatible, carbon facilitates covalent anchoring of specific biological species such as enzymes, proteins and DNA molecules. Glassy carbon electrode (GCE), pencil graphite electrode (PGE) and screen printed carbon electrodes (SPCE) are commonly used biosensor electrodes [26, 27].

GCE is a non-graphitic carbon, combining glassy and ceramic properties with those of graphite, synthesized by high temperatures pyrolysis of certain polymeric precursor above 2000 °C. GCE is chemically stable and compared to other types of carbon materials it possesses rather low reactivity, low oxidation rate, high chemical inertness, high hardness, impermeability due to very small pore sizes, and good electrical conductivity which make it a competitive inert, conductive electrode.

With customizable conductive inks and three-dimensional (3D) printing technology, more reproducible than simpler drop-casting methods, SPCE benefit from simplicity, cost-effectiveness, and suitability for mass production. Also, SPCEs enable simple integration and desired portability [28]. PGEs, although more fragile than SPCEs, are also a readily available and disposable low-cost carbon-based device. Screen printed pencil graphite electrodes can be conveniently designed in a similar shape to SPCE [29]. However, establishing clean electrodes before any surface modification is particularly important because they are subject to unintentional adsorption of impurities.

Biological compound detection with bare electrodes is difficult due to poor responsiveness and high over-potentials. Hence, electrode surface modification seeks to improve the electrochemical characteristics, either by pretreatment [30] or by chemical modification (e.g. electrooxidation or electroreduction). Prasad et al. pretreated bare-SPCE by applying a potential at 2.0 V for 300 s in pH 7.4 PBS (phosphate buffer solution) when investigating oxygen functional groups and edge plane sites on SPCE for the determination of dopamine (DA), uric (UA) and ascorbic (AA) acids [31]. The edge plane sites were the principal location for electron transfer arising from oxygen functionalities on active sites. Electrode pretreatment could introduce more edge planes and oxygen groups in the lattice. Unlike bare-SPCE or oxygen plasma treated SPCE, electrochemically pretreated SPCE* could simultaneously detect DA, UA and AA [31]. Edge plane sites on the surface improved electron transfer kinetics and increased resistance to surface passivation. In contrast to SPCE and PGE, bare-GCE contained a significant number of edge plane sites, confirmed by Raman spectroscopy. However, bare-GCE have low oxygen content and despite the introduction of oxygen functionalities by treatment in acidic solution [27] or plasma [32], the efficiency of such modified GCE for electrochemical biosensors remained debatable. Electrode topography also directly influenced analyte detection sensitivity. For example, high electrochemical reactivity of PGE, from an irregular surface morphology, increased the active surface area of the electrode (e.g., 0.255 cm^2^ for PGE compared to 0.0951 cm^2^ for carbon paste electrode) [26]. In the design of an electrochemical label-free biosensor detecting microRNA-125a, Yammouri et al. used PGE modified with several carbon nanomaterials, including carbon black (CB), multiwalled carbon nanotubes (MWCNT) and graphene oxide (GO). Their lowest LOD was 10 pM (1 pg/mL) obtained using PGE modified with CB providing a linear range between 1 nM and 2 μM [33]. Exploratory use of pretreated bare carbonaceous electrodes in electrochemical biosensors for healthcare applications introduced key questions; could we detect molecules with high efficiency on surface-modified GCE, PGE or SPCE and would this surpass biosensors based on alternative 2D nanomaterials?

### Electrode fabrication: enhancing bare electrodes with nanomaterials

Nanomaterials are very appealing materials for development of innovative future biosensors. Exceptionally small size and high surface area to volume ratios permit intimate interaction with target biomolecules [34, 35]. By comparison, sensors uniquely based on bare GCE, PGE or SPCE show limitations. Carbon nanomaterials provide advantages for electrochemical carbon based nanosensors in pharmacology and biomedicine [36]. The use of fullerenes and carbon nanotubes in electrochemical biosensors [37] may be compromised by a tendency for zero- (0D) and one- (1D) dimensional nanostructures to self-aggregate. In contrast, two-dimensional materials have a higher surface to volume ratio offering better stability for molecular interactions [38]. Graphene, a 2D hexagonal carbon structure arranged from sp^2^ hybridized carbon atoms, is the fundamental carbon precursor for graphitic materials with other dimensionalities; folded into 0D fullerenes, rolled into 1D CNTs, or stacked into 3D graphite. Graphene’s unique properties include an atypically high specific surface area (2600 m^2^/g), mechanical strength (130 GPa) and electrical conductivity reaching 6000 S/cm in its pristine form. Various approaches have aimed to synthesize graphene, such as chemical vapor deposition (CVD), chemical exfoliation methods or physical vapor deposition (PVD) [39]. It has to be noticed that PVD is a relatively new field to grow 2D materials, and graphene produced by this technique exhibit excellent electrochemical properties [40]. Nowadays, the chemical oxidation of graphite followed by exfoliation in aqueous solvents to produce GO and/or its subsequent reduced form remains a popular pathway for generation of ultraperformance graphene electrochemical biosensors [41].

Nanomaterials used in biosensing vary in morphology (size, shape), mechanical, optical, electrical and chemical properties, biocompatibility and stability [42]. Diverse synthesis methods provide materials with various functionalizations and crystallinity, yet exactly how those parameters influence the electrochemical biosensing efficiency is rarely discussed. For elucidation of structure/reactivity relationships of electrochemical electrodes we take graphene as a benchmark.

## Morphology, disorder and defects influence electrical conductivity of graphene: a critical aspect for biosensing

### The variety of graphene, graphene composites and issues of nomenclature

**Fig. 1:**
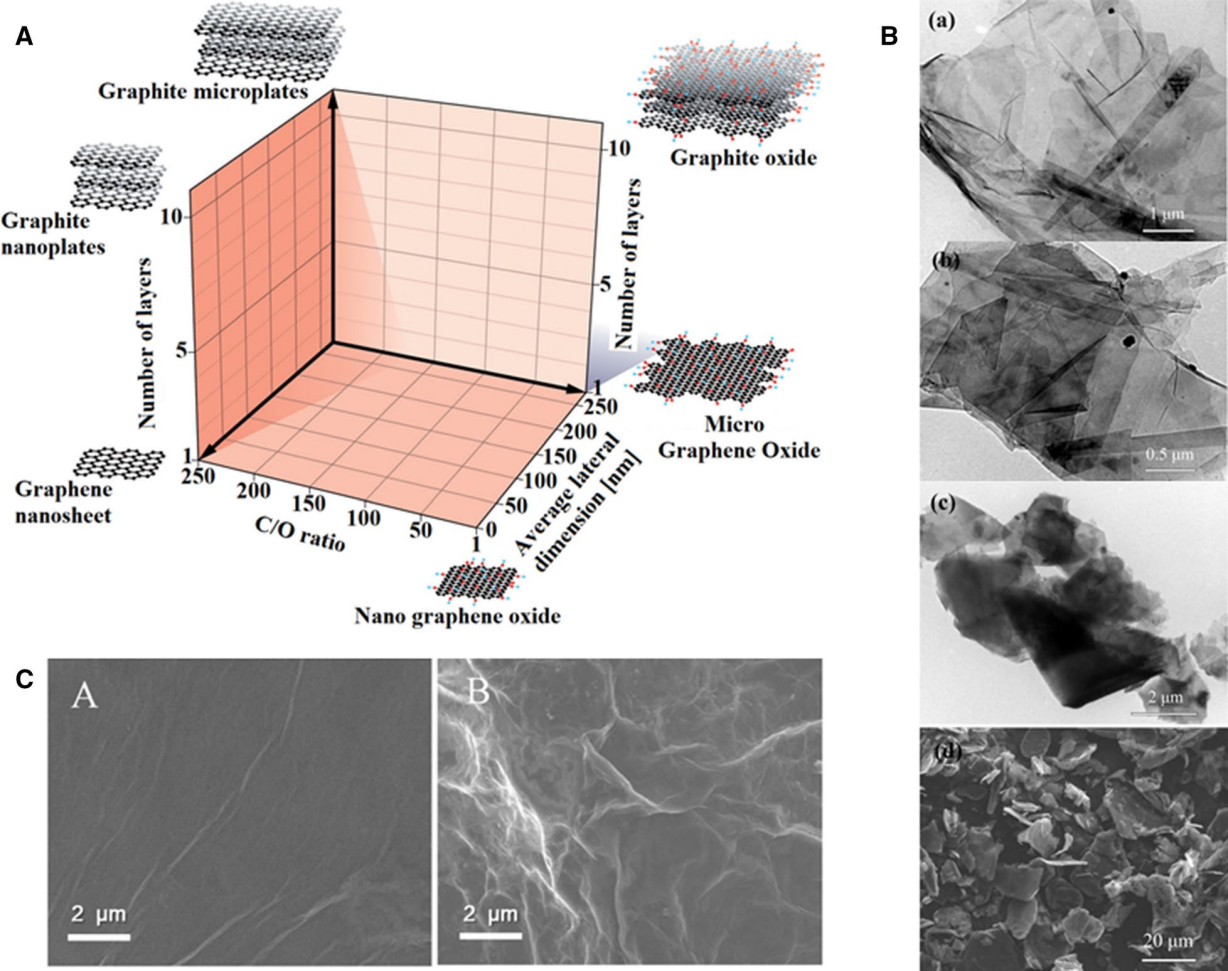
Morphology of various graphene materials. **A** Classification grid for the categorization of different graphene types [43]. **B** TEM images of graphene nanoplates (1–5 nm), graphene nanoplates (5–20 nm), micro-graphite, and SEM image of natural graphite (top to bottom) [50]. **C** SEM images of GO (**a**) and ERGO (**b**) [118]

In the broadest sense, graphenic nanomaterials encompass a variety of carbon sheets with different number of layers (single, few, multi) and a range of lateral sizes, oxygen content and functional groups, thus the term “graphene” warrants careful application. Recently, Wick et al. categorized different graphene types according to thickness, lateral dimension and atomic carbon/oxygen (C/O) ratio [43] (Fig. 1a), bringing more clarity in this field. Establishing standardization and nomenclature will be especially important to avoid misunderstanding between researchers, industry and authorities in the demanding context of healthcare applications. Key questions concern the influence of the layers, lateral size and C/O ratio on the electrochemical detection of molecules. Depending of the carbon source (e.g. graphite, CNT, SiC, agricultural waste) and synthesis method, various graphene forms with distinct qualities can be obtained, such as multilayer (ML) graphene [44, 45], graphene nanoribbons after unzipping the CNTs [46], graphene oxide, reduced graphene oxide (rGO) and graphene quantum dots (GQDs) [47]; plus functionalized graphene with various additional groups (COOH, NH_2_, etc.) [48, 49]. Scanning electron microscopy (SEM) reveals graphite and graphene nanoplatelets (Fig. 1b) as short piles of platelet-like graphene sheets, identical to those in the walls of CNTs, but in a planar structure [50]. GO and rGO modified GCE by drop-casting and electrochemical reduction, respectively, show different surface morphology (Fig. 1c). Reduced graphene oxide usually has an increased number of wrinkles which was found highly beneficial for the design of electrochemical biosensor with enhanced surface area. Introducing additional complexity, graphene can be mixed with nanoparticles or doped with various heteroatoms (e.g. N, B and S) [51], fabricated in 3D architectures or in a plethora of composites (e.g. foams), many applicable to electrochemical biosensing [52, 53].

### Engineered defects in graphene; understanding their role in electrochemistry

**Fig. 2:**
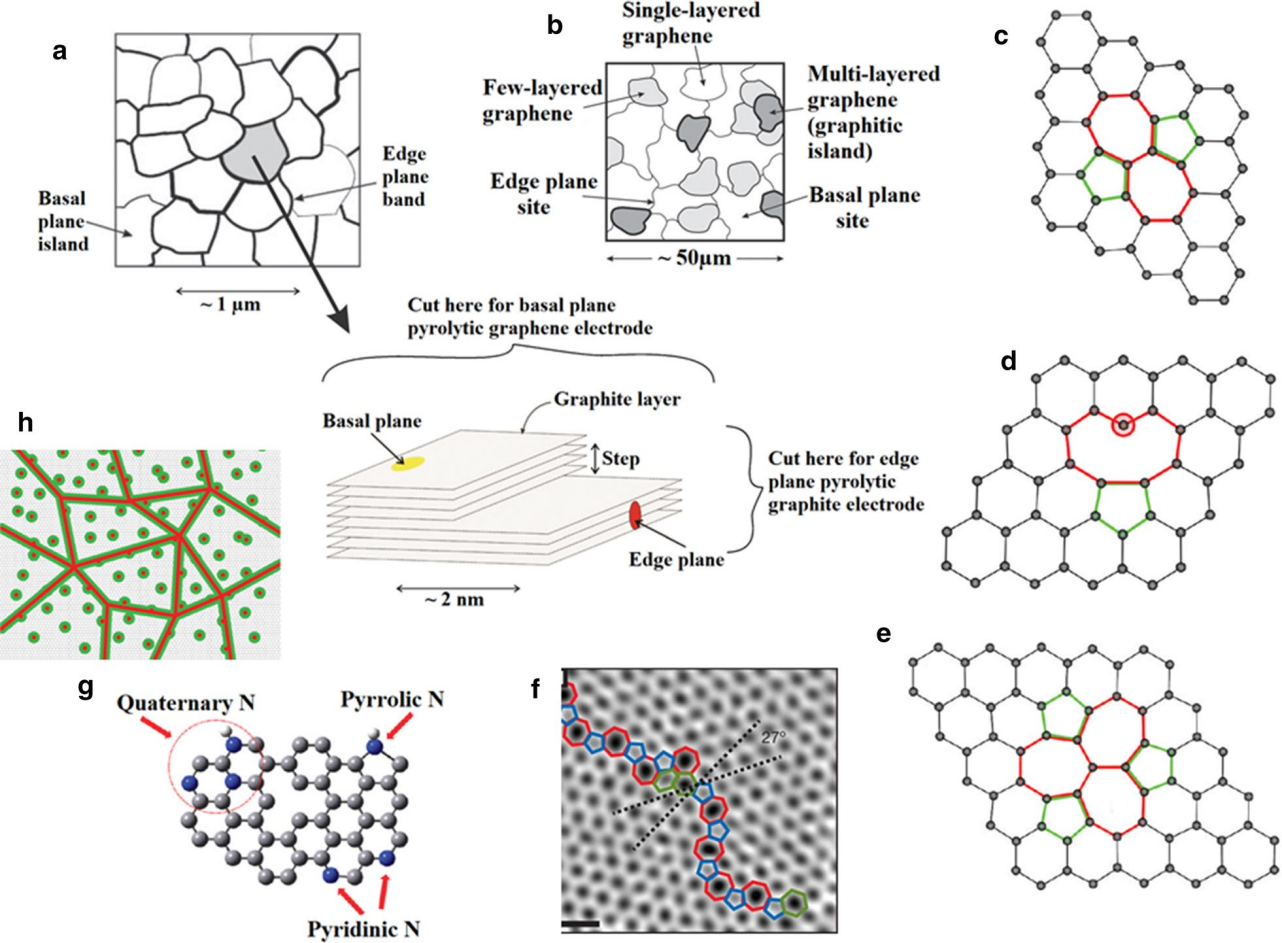
Versatility of defects in graphene. **a** Schematic representation of a HOPG surface showing discrete basal plane and edge plane islands. Side on view of the HOPG surface highlighting its basal plane and edge plane like-sites/defects which exhibit contrasting behaviours in terms of electrochemical activity, where electron transfer kinetics of the latter are overwhelmingly dominant over that of the former which in comparison are relatively (electrochemically) inert. Reprinted from [54] with permission of The Royal Society of Chemistry. **b** An underlying graphene surface with few- and multi-layered graphitic islands, indicating the basal and edge plane electron transfer sites; notice the electrode surface is akin to a HOPG surface. Reprinted from [54] with permission of The Royal Society of Chemistry. Graphene atomic structure as obtained from density functional theory (DFT) calculation for: Stone–Wales defects **(c)**, single vacancy defect **(d)**, and multiple vacancy defects **(e)** in graphene. Reprinted with permission from [146]; copyright 2010 American Chemical Society. **f** Aberration-corrected annular dark-field scanning transmission electron microscopy (ADF-STEM) of line defects in graphene; scale bars: 5 Å. Reprinted with permission from [147]; copyright (2010) Nature Publishing Group. **g** Graphene in-plane heteroatom substitution defect model: nitrogen defects. Reprinted with permission from [148]; copyright (2010) American Chemical Society. **h** Illustrations of a graphene sample containing both point and line defects. The red regions define the structurally damaged area (S-region), and the green circles and lines are the activated area (A-regions) where the D band is active (Reprinted with permission from [149]; copyright 2017, IOP Publishing)

The electrochemical electrode response to reactants is mainly dependent on electron transfer kinetics and available surface area. The rate of heterogeneous electron transfer (HET), i.e. the transfer of electrons from/to electrode to/from molecules, is also correlated to the electrodes’s density of electronic states (DOS) and surface chemistry (number of defects, functional groups, impurities etc.). Introducing disorder/defects and roughness achieves a higher DOS, improving the HET of an electrode. It is established that the existence of edge plane sites/defects on graphitic materials improves the electron transfer processes [54] (Fig. 2a), occurring about 10^6^ times faster at defects or edge-like sites versus basal or defect-free planes [55]. Faster electron dynamics at graphene edges result in excellent capacitive and electrocatalytic properties [56, 57] with four-fold specific capacitance and doubled current density found at the edge instead of the basal-plane. The HET rates of edge and basal planes of graphite are dependent on the type of inner and outer-sphere redox system used. For example, in the case of [Ru(NH_3_)_6_]^3+/2+^, the HET for both planes is similar, whereas for [Fe(CN)_6_]^3−/4−^ it is dramatically different since the edge-plane activity is faster [55]. For the [Ru(NH_3_)_6_]^3+/2+^ redox system with an outer-sphere electron transfer mechanism, the charge transfer rate is mostly affected by the electronic properties of the electrode, in particular its DOS near the Fermi potential. In contrast, [Fe(CN)_6_]^3−/4−^ represents an inner-sphere redox probe, thus its electron transfer kinetics is dependent on both surface microstructure and DOS.

Although single-layer pristine graphene of perfect 2D crystal structure has promising applications, its electrochemical activity suffers from an “Achilles’ heel” lack of an edge-plane band or significant surface defect densities [54]. Nonetheless, graphene acquires significant electrochemical properties when in few or multilayers structures (Fig. 2b). Existing defects (kinks, vacancies, steps) on the edge planes of epitaxial graphene (EG) generate localized edge states that result in high DOS near the Fermi level and increased electron transfer kinetics [58].

To monitor electrode modifications and, in particular, to access the presence of defects, Raman spectroscopy is a powerful, fast, non-destructive method, sensitive to both the electronic and phonon properties of the sample surface. Carbonaceous materials, characteristically generate a key quantifiable ratio between the spectrum’s D peak at approximately 1350 cm^−1^ and G peak at circa 1580 cm^−1^ [59]. The D peak corresponds to the breathing vibration of carbon in both sp^3^ bonds and sp^2^ rings on the edge-plane, while the G peak corresponds to the stretching vibration of carbon in both sp^2^ chains and rings in the basal-plane. The ratio of the D peak around 1350 cm^−1^ and G peak around 1580 cm^−1^ provides an estimate of the density of defects [60]. For the electrode pretreated by Prasad et al., the enhanced edge planes defect sites could also be confirmed by higher intensity ratios between D and G peaks in the Raman spectra [31]. For N layered graphene, the stacking order between layers has a significant influence on the band and interlayer phonon properties [60]. Meanwhile, a more disordered or defective structure induced frequency shifts and an increase in the linewidth of the Raman bands. Edges naturally exist in graphene whatever the technique used for synthesis, and they represent a kind of defect because the translational symmetry is broken. The lack of edge plane defects of pristine graphene can be highlighted by the absence of the D peak [43]. The high I(D)/I(G) ratio in GO confirms that its lattice is distorted and has a large amount of sp^3^-like defects caused by the oxidation process. The 2D peak between 2650 and 2700 cm^−1^ is, as the G peak, also characteristic of sp^2^ hybridized carbon–carbon bonds in graphene, and is extremely sensitive to defects, as well as, doping, thickness of graphene and nature of the substrate holder [60, 61]. The absence of 2D mode in GO primarily indicated a fully-disordered sp^2^ bonding structure mainly caused by functional groups. In contrast to mechanically exfoliated graphene, where the stacking order is well controlled, and 2D peak is a benchmark for the determination of the number of layers (NL), in the case of GO, the 2D peak is not related to NL. Few studies have been reported in this sense. Toh et al. has proposed an ingenious microinjection-micromanipulator system in order to estimate the heterogeneous electron transfer rate (k^o^) in function with the number of layers for IrCl_6_^2−/3−^ probe [62]. On Si/SU8 substrate, the k^o^ value augments with increasing NL: 3.08 × 10^−3^, 8.1 × 10^−3^, 1.06 × 10^−2^ cm s^−1^ for mono, few and multilayer graphene in the basal planes, respectively. On the edge/step between the few and multiplayer graphene flakes, the value was 9.88 × 10^−2^ cm s^−1^, showing a paramount importance to have edge plane defect sites in biosensing. As a reference, k^o^ value was 1.93 × 10^−2^ cm s^−1^ for graphite. However, the substrate is believed to influence electrochemical properties and the k° value. As aforementioned, the synthesized graphene has a panel of morphology, and graphene tends to preferentially form aggregates to minimize the presence of edge plane sites. Smart approaches should be employed to avoid the morphological aggregation process [63]. Akhavan et al. has compared the DPV electrochemical activity of GCE, graphite, GO and rGO nanosheets, GO and rGO nanowalls (NWs). Due to the extremely sharp edges vertically aligned on GCE, the GO NWs and rGO NWs show an enhanced electrochemical reativity of the four free DNA bases, single-stranded (ss) DNA and double-stranded (ds) DNA [64].

**Fig. 3:**
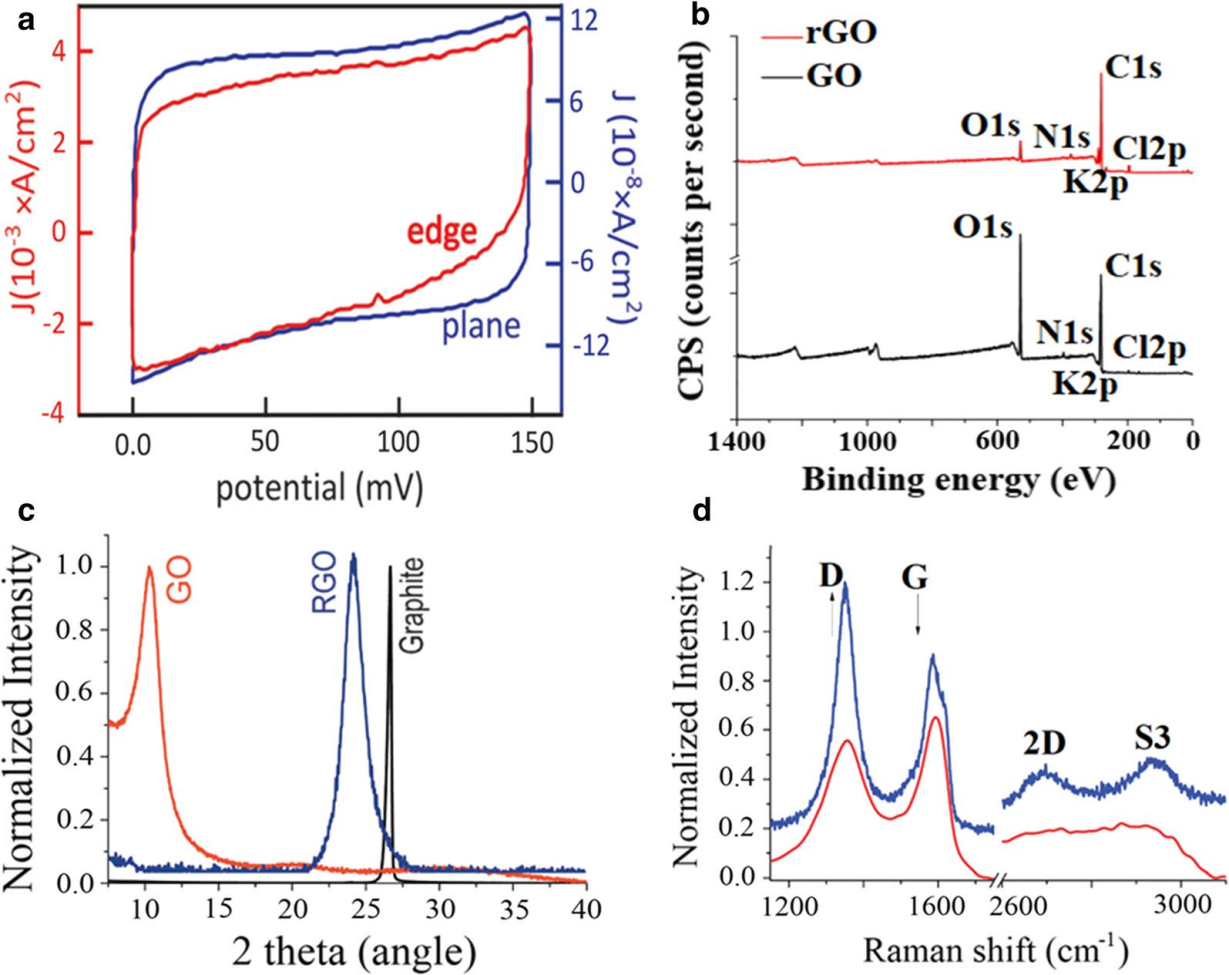
Structural properties of various graphene materials. **a** CVs of the basal plane (blue) and a graphene edge (red) in an aqueous solution of phosphate-buffered saline. Reprinted with permission from [57]. **b** XPS spectra of GO and rGO [135]. **c** XRD pattern of graphite (black), GO (red) and rGO (blue) [74]. **d** Raman spectra of GO (red) and rGO (blue) [74]. After reduction the intensity ratio between D and G (I(D)/I(G) increased

Synthesized graphene and GO retain desirable defects [65, 66] (Fig. 2c–h) that can be divided into two categories; foreign adatoms and substitutional impurities. Defects can be introduced by different ways, such as ion-implantation, plasma treatment, functional groups. Substitutional impurities e.g. nitrogen, can form three chemical bonds and so take the place of carbon atoms in the graphene lattice. Graphene defects change the length of the interatomic valence bond and orbital, thereby altering its electrical properties. Point and single vacancy defects, unavoidable in current preparation methods, produce a decrease in the conductivity of graphene. Intrinsic defects aside, conductivity can also decrease due to the existence of oxygen groups and square resistance in GO can reach more than 10^12^ Ω. In contrast to oxygen, addition of different atoms such as nitrogen or boron, improved the conductivity of graphene [67]. In comparison to basal planes, the electrochemical activity of the graphene at its edge defective site is higher with an electron-transfer current from CV curves enhanced up to four orders of magnitude (Fig. 3a) [56]. Defects in GO can be either permanent or transient, the latter can be healed after a reductive restoration [66]. Self-healing graphene oxide/polymer composites are of great interest in the biomedical sensing field.

### Comparing defect influence on graphene oxide versus reduced graphene oxide electrodes

GO is generally prepared by oxidation of graphite with subsequent exfoliation by sonication to obtain colloidal suspensions of mono-, bi-, or few-layer graphene oxide sheets [68]. Sonication effects on the mechanical properties and yield of GO is reported by various groups [69–72]. Helping control oxidation levels on the GO surface, Zeng et al. oxidized a graphite electrode using a one-step electrochemical approach by applying a positive potential between 0 and 3.0 V at 50 mVs^−1^ in 0.025 M phosphate buffer solution. Oxidation of graphite occurs at around 1.4 V and graphene oxide nanosheets could be obtained with good coverage for only 2–5 CV scans [73]. The surface of natural graphene is hydrophobic with little dispersibility in most solvents, whereas GO has hydroxyl and epoxide groups on the basal plane, plus carbonyl and carboxyl groups positioned at the sheet edges providing hydrophilicity for water dispersibility. By X-ray diffraction (XRD) was indicated that sonication increases the interlayer distance of graphene sheets confirming a good exfoliation effect [74]. Sehrawat et al. show using high-resolution-transmission electron microscopy (HRTEM) an obvious increase of the GO sheet transparency with an increase of ultrasonication time [75]. Subjected to sonication parameters of 40 kHz and 400 W, the size of the GO sheets may decrease sharply within the first hour of a sonication yet then remained relatively unchanged after 5 h of sonication, with a typical sheet surface area less than 200 µm^2^ [70]. Disaggregated into small flakes, GO sheets tend to restack together [70]. Dispersed GO is typically characterized by a 2:1 C/O ratio [76]. Unlike pristine graphene, defects on the GO surface greatly influence sensing performance by providing sites for strong interactions with charged species [77]. However, although GO surface groups help bind molecules avidly, it is less conductive, thus a reduction process is often performed to reach a compromise between reactive groups and conductivity.

Reduced GO can evolve from various reduction approaches including chemical, thermal, hydrothermal, microwave, microbial/bacterial, photo-chemical/photothermal, and electrochemical methods [78]. Reduction converts sp^3^ to sp^2^ carbon domains mediating important changes in the physico-chemical properties of GO (e.g. higher C/O atomic ratio, decrease of interlayer distance, Fig. 3b, c). The main reduction methods explored are chemical, thermal, hydrothermal and electrochemical [79]. For chemical reduction methods (CR), hydrazine hydrate (N_2_H_4_·H_2_O) is the most used agent, although other reductants have been employed with good efficiency [68]. Thermal reduction (TR) is generally performed by heating GO above 1000 °C [68]. However, some recent studies reported good reduction by annealing using temperature below 350 °C [80, 81]. Hydrothermal reduction (HR) is a green method in comparison to CR and TR because the use of moderate temperature and pressure in aqueous solution [82]. Amongst the numerous reduction methods, the electrochemical approach is a fast, easy, economic, scalable, and environmentally friendly route [62].

Typically, reduction of GO is accompanied by smaller sp^3^ domain sizes compensated by more sp^2^ domains, indicated by a higher ratio between the intensity of D and G peaks and a narrower D band in rGO compared to GO in the Raman spectra (Fig. 3d). Carbon material versatility reflects strong interdependence between physical properties and the ratio of sp^2^ (graphite like) to sp^3^ (diamond like) bonds. A three stage amorphization trajectory model has been proposed to describe the introduction of a series of defects in a perfect graphene sheet, transitioning from: (1) graphite to nanocrystalline graphite (nc-G); (2) nc-G to amorphous carbon (a-C); a-C to tetrahedral amporphous carbon (ta-C) [83]. The intensity ratio I(D)/I(G), an indicator of carbon network order or disorder, has been used to estimate lateral crystallite size L_a_ according to a Tuinstra-Koening relation. Although this ratio reportedly varies in an inversely proportional manner to L_a_ [84], this did not strictly concord with the observations of Ferrari et al., since one could expect presence of sp^3^ as well as sp^2^ domains during progressive reduction of graphene [83, 85]. Rather, the evolution of I(D)/I(G) with the crystallite size was in perfect agreement with predictions for the stage 2 transition from nc-G to a-C [83], while the Tuinstra-Koening relation was only valid for the stage 1 graphite to nc-G trajectory. Reduction of GO may provide abundant structural defects and improve drastically the electrochemical activity of bare and GO modified electrodes. However, contrary to convention, some have reported that GO had a better CV signal, even though GO itself should be less conductive [86].

The improved electrical conductivity from partial restoration of π-conjugated sp^2^ structure, with more chemically reactive defective sites make rGO a favoured graphene material for the detection of DNA. Direct comparison of GO *vs* rGO for DNA adsorption measured by fluorescence spectroscopy revealed a better sensing ability for rGO due to a richer sp^2^ carbon surface allowing better π–π stacking with DNA bases [87]. Scanning electron microscopy images of electrochemically reduced GO reveal an irregularly crumbled and sheet-like structure with an increased density and thickness proportional to the number of cycles [88]. Consequently, this increased the effective surface area and improved the conductivity. Based on the Randles–Sevcik equation, GO has a higher active surface area with a better electrochemical reaction ability [89–91]. It is possible to reversibly control the introduction of defects at graphene boundaries [92]. Monitoring the oxygen content and fraction ratio of sp^2^ to sp^3^-hybridized carbon atoms can be used to tune the carrier mobility by over 12 orders of magnitude and though residual oxygen in rGO prevents carrier mobilities from equating to pristine graphene values, high electron mobilities over 1000 cm^2^ V^−1^ s^−1^ have been recorded in thin rGO films [93]. However, rGO seems chemically less stable than graphene and GO [94–96]. It is therefore clear from recent studies that each graphene material type has advantages and drawbacks with debate as to which one could prove best for biosensing.

GO and rGO could be assembled onto the electrode by various techniques such as drop-casting, dip-coating, spray coating and layer-by-layer deposition [1]. A film forms through π–π electronic interaction between graphene and the bare electrode [97]. The electrochemical reduction (ER) of GO could be realized in various electrolytes. Two different pathways are generally undertaken; the one-step path involves the direct electrochemical reduction of graphene oxide in colloidal solution by a supporting electrolyte on the electrode. The two-step method involves the pre-deposition of GO onto the electrode before its reduction using a conductive electrolyte on the electrode. Table 1 shows recent protocols used for the preparation of graphene modified GCE, PGE and SPCE.

**Table 1:** Platforms using rGO or GO on glassy carbon electrode, pencil graphite electrode and screen printed carbon electrode

Electrode platform	GO assembly method	Reduction of GO	Supporting electrolyte	pH of electrolyte	Applied potential	Electrochemical time/cycle	References
GCE/rGO*	Drop-cast 10 µL GO (2 mg/mL)	Electrochemical	Na-PBS	4	− 0.9 V	2000 s	[98]
GCE/rGO*	Drop-cast GO (1 mg/mL)	CV	N_2_-purged PBS	7	From 0.0 to − 1.5 V	15 cycles	[99]
GCE/rGO	Drop-cast 5 µL rGO	Chemical (hydrazine)	PBS	7	From − 1.5 to + 1.1 V	30 cycles	[100]
PGE/rGO*	N/A	CV	GO suspension (1 mg/mL)	8.5	From 0.0 to − 1.0 V	10 cycles	[101]
PGE/rGO*	N/A	Electrochemical	GO suspension (0.12 mg/mL)	N/A	− 1 V	260 s	[102]
PGE/GO	Immerse PGE in 100 µL GO (400 µg/mL) for 15 min	N/A	N/A	N/A	N/A	N/A	[86]
SPCE/rGO*	Drop-cast 5 µL GO (0.1 mg/mL)	CV	PBS	7	From 0.0 to − 1.5 V	Until a constant current was achieved	[103]
SPCE/rGO*	Drop-cast 5 µL GO (0.3 mg/mL)	CV	N_2_-purged KCl	N/A	From 0.0 to − 1.4 V	10 cycles	[104]
SPCE/rGO*	Drop-cast 100µL GO/APBA (1 mg/mL)	CV	Na_2_SO_4_	N/A	From − 1.2 to + 1.2 V	40 cycles	[105]

* Reduced graphene oxide is part of a composite-based platform

### Sensors comparing oxidized graphene and reduced graphene oxide

Applying a CV potential of up to 2.0 V for 500 s to oxygenated epitaxial graphene in PBS (anodization process), a mixture of DNA bases could be detected simultaneously, without a pre-hydrolysis step. dsDNA was discriminated from ssDNA with better sensitivity than when using GCE and boron doped diamond. Moreover, mixtures of biomolecules (AA, DA and UA) were treated as individual peaks [58]. This was associated with both an increase in the electron transfer rate (0.0101 cm/s) and edge plane defects. The latter were correlated to both an increase in D peak intensity (higher I(D)/I(G) ratio) and oxygen content. The results were supported by both qualitative (using both [Fe(CN)_6_]^3−/4−^ and [Ru(NH_3_)_6_]^3+/2+^ redox probes) and quantitative analysis (reversible process through Randles–Sevcik and Nicholson equations). An ability to controllably change the anodization time, served to accurately tune the graphene defect density. It was clearly shown that anodized EG at 200 s had a slower HET rate constant than the one anodized at 500 s. The latter had more defective sites, a sharper redox [Fe(CN)_6_]^3−/4−^ peak separation and, higher capacitance in potassium chloride (KCl) at 0.025 V.

**Fig. 4:**
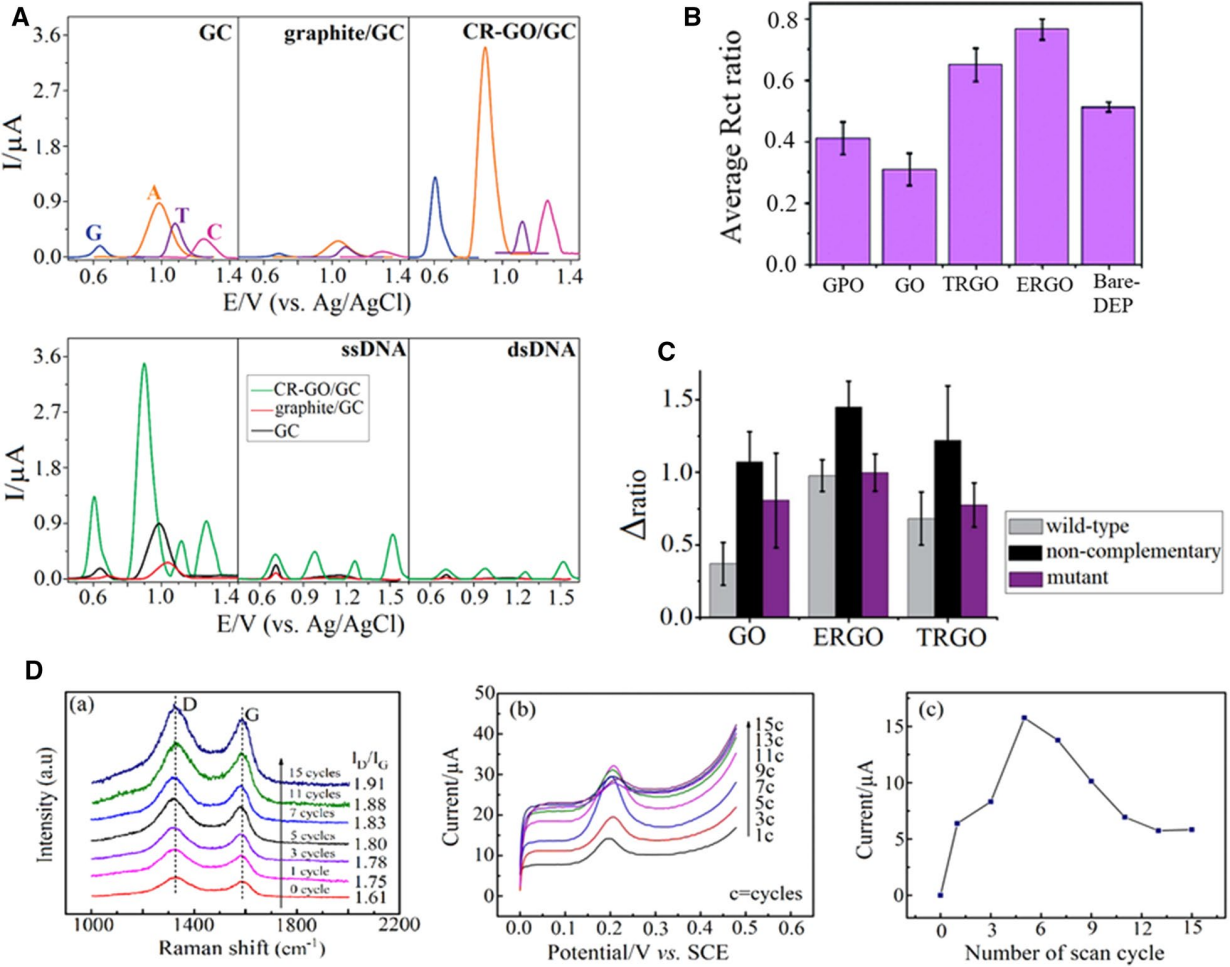
Comparison of bare electrode, graphite electrode and various graphene electrodes on electrochemical sensing. **A** (a) DPVs at the GC electrode for G (blue), A (orange), T (violet), and C (magenta), respectively; (b) DPVs at the graphite/GC electrode for G (blue), A (orange), T (violet), and C (magenta), respectively; (c) DPVs at the CR-GO/GC electrode for G (blue), A (orange), T (violet), and C (magenta), respectively; (d) DPVs for a mixture of G, A, T, and C at CR-GO/GC (green), graphite/GC (red), and GC electrodes (black); (e) DPVs for ssDNA at CR-GO/GC (green), graphite/GC (red), and GC electrodes (black); (f) DPVs for dsDNA at CR-GO/GC (green), graphite/GC (red), and GC electrodes (black). Concentrations for different species (a-f): G, A, T, C, ssDNA or dsDNA: 10 μg/mL. Electrolyte: 0.1 M pH 7.0 PBS. Reprinted with permission from [106]; Copyright 2009 American Chemical Society. **B** Histogram representing a comparison of impedimetric signals on GPO, GO, TR-GO, ER-GO and bare DEP electrode. Signal is represented as average Rct ratio ((Rct protein − Rct blank)/(Rct aptamer − Rct blank)). Error bars correspond to triplicate experiments. All measurements were performed with 10 mM K_4_[Fe(CN)_6_]/K_3_[Fe(CN)_6_] in Tris buffer solution (pH = 8.2) at room temperature. Reprinted from [107] with permission of The Royal Society of Chemistry. **C** The comparison of impedimetric response on graphene oxide, ER-GO and TR-GO recorded after hybridization with wild-type (grey), mutant (purple) and non-complementary (black) target DNA sequences. The signal is represented as Δratio = Δt/Δp, Δt = Rct, target-Rct, blank, Δp = Rct, probe-Rct, blank. Standard deviations correspond to triplicate experiments. Reprinted from [108] with permission of The Royal Society of Chemistry. **D** (a) Raman spectra of ERGO film at different CV cycles; (b) CV curves containing 30 µM isoniazid (INZ) at different reduction cycles, and (c) line chart of the relationship between reduction cycle number and peak current response towards INZ [118]

Zhou et al. compared the electrochemical sensing ability of CR-GO/GCE (chemically reduced GO) with graphite/GCE and GCE with a different morphology (Fig. 4a) [106]. Their DPV measurement indicated that CR-GO/GCE had a better sensing performance to detect DNA nucleotides, ss and ds. Moreover, single-base mismatches (G→A or C→T mutations) within single-nucleotide polymorphisms (SNPs) and multiplexed DNA nucleotides could be accurately detected using CR-GO/GCE. Other molecules such as glucose or β-nicotinamide adenine dinucleotide (NADH) were also detected by such methods. The enhanced electrochemical properties could be attributed to its single-sheet nature, high conductivity, a better reaction ability (apparent electrode area A ~ 0.092 cm^2^), lower charge transfer resistance (160.8 Ω), antifouling properties and a higher density of edge-plane defect sites (I(D)/I(G) ~ 1.38). Edge-plane defects provided numerous active sites valuable for accelerating electron transfer between the electrode and species in solution. However, it was clear from an I(D)/I(G) of graphite/GCE close to 0 [27], that defects were not the only contributing factor for fast electron transfer. Unique characteristics of CR-GO/GCE presumably made the electron transfer easier.

One of the most informative direct comparisons was undertaken by Báez et al., who compared the electrochemical performance of graphene oxide reduced by chemical (CRGO), thermal (TRGO), hydrothermal (HRGO), and electrochemical (ERGO) methods taken from a similar GO source [79]. The reduced GO samples had obvious morphological differences, with specific surface areas, oxygen contents, and electrochemical activity (Fig. 4b). TRGO had the best sensitivity for the detection of guanine by DPV measurements. The performance of TRGO likely reflected its dramatically different morphology (highest porosity and surface area), highest number of electroactive sites (more significant CV current), and good electrical conductivity (one of the highest C/O ratio). However, in contrast CR-GO/GCE, it was unlikely to be related to common defects activated in the Raman spectra, since TRGO had the lowest I(D)/I(G) ratio. Also, although their electron transfer could be fast, it was not the fastest with regard to the redox peak separation. In contrast, by comparing graphite oxide, GO, TRGO and ERGO dried-absorbed on DEP-chips (disposable electrical printed chip), GO provided the best sensitivity for the detection of the important blood coagulation protein thrombin [107] (Fig. 4c). From similar experimental comparisons, in the same research group, GO on DEP-chips was the best candidate for the detection of SNPs correlated to Alzheimer’s disease [108] (Fig. 4d). Despite convincing experimental evidence using only impedimetric measurements, no surface and structural analysis was provided. Giovanni et al. gave a possible explanation of a better sensitivity for GO (e.g. presence of defects and oxygen groups) claiming agreement with the earlier studies of Lim et al. [58]. However, a comparison of XPS spectra clearly indicated that the C/O ratio of GO for each study was drastically different. Examples of such differences between laboratories using broader electrochemical methods for detection and with greater emphasis on the quantitative analysis (spectroscopy, calculation of charge transfer resistance, etc.) could bring more clarity to this field.

### Functionalization and composite materials broadening the scope of electrochemical biosensor tunability

Structurally modifying graphene through chemical and physical functionalization methods revealed the numerous possibilities for tuning its structure [109]. By performing EIS measurement, Bonnani et al. showed that chemically reduced GO with perpendicularly grafted carboxyl groups, mainly at edge sites (CRGO-COOH), dramatically outperformed the DNA sensing performance of GO as a result of a more efficient immobilization of the probes [48]. The probe density estimated from chronocoulometry was 2.41 × 10^13^ and 3.76 × 10^13^ molecules cm^−2^ for GO and CRGO-COOH, respectively. Interestingly, COOH groups on CRGO did not change its electrochemical response significantly (CV and EIS curves) meaning no significant damage was introduced during grafting. This seemed in good agreement with the fact that the grafted COOH groups did not introduced many defects in the structure as indicated by Raman measurements [110, 111].

The concept of combining graphene with other materials to enhance its electrochemical properties is very compelling, taking advantage of synergistic effects for enhanced biosensors. Several methods have been developed for the preparation of less aggregated and more stable graphene electrochemical biosensors from graphene-based composites. Less graphene aggregation not only provides a high active site density but also increases the surface area and porosity, both extremely beneficial for the anchorage of biological molecules. For example, graphene has been found to be an excellent 2D surface to incorporate Au, Pt and Pd nanoparticles for applications in energy, catalysis and sensing, by way of providing improved charge transfer resistance and peak current intensities. Nonetheless, many challenges remain to be resolved [112]. Nanoparticles are often electrodeposited on the surface of graphene. For example, PtAu nanoclusters could be deposited on rGO in aqueous solution containing 0.2 M H_2_SO_4_, 0.5 mM HAuCl_4_ and 0.5 mM H_2_PtCl_6_ with a deposition time and potential of 400 s and − 0.2 V, respectively [97]. Gold nanoparticles (AuNPs) modified graphene electrodes are probably the most widely used for sensing devices due to their unique optical and electronic properties, stability, low cytotoxicity and relative ease of synthesis with a typical size ranging from 3 to 200 nm. This hybrid material could be used in almost any domain of sensing, ranging from optical fluorescence to electrochemical approaches. For example, by modifying PGE with AuNPs, Mandli et al. designed a highly sensitive and selective electrochemical microRNA (miRNA) biosensor with a LOD of 100 pM and a linear range from 200 to 388 nM [113].

Nowadays, complex graphene composites biosensors are elaborated but quantitative analyses correlating how charge electron transfer affects the biosensors have yet to be completed. In summary, a good electrode needs to balance conductivity and defect density [114]. Also, it is clear that the surface area, morphology, chemistry, functionalization and newly developed hybrid graphene materials play an important role towards high efficient biosensors. Table 2 highlights studies concerning the structural, chemistry and electron transfer properties of electrochemical biosensors based on graphene and graphene composites.

**Table 2:** Influence of morphology, defects and conductivity on electron transfer properties of electrochemical biosensors based on graphene and graphene composites

Electrode platform	Morphology	Carbon to Oxygen ratio [%]	I_D_/I_G_ ratio	Charge transfer resistance (Rct [Ω])	Surface area [cm^2^]	Peak-to-peak potential (ΔEp [mV])	Heterogenous electron transfer rate constant [cm/s]	Capacitance (µF/cm^2^)	References
Gr-HPHT diamond	Island-like surface structure	N/A	0.29	400	0.0183	N/A	N/A	N/A	[115]
Thi-rGO/GCE	Flake-like shape with slight wrinkles	1.654	Higher than GO	33.41	N/A	N/A	N/A	N/A	[116]
Au-rGO-AuPtNP	Bended sheets of graphene	N/A	1.02	N/A	N/A	80	N/A	N/A	[117]
ERGO-GCE	High degree of wrinkles	N/A	1.80	Decreases compared to GO	N/A	N/A	N/A	N/A	[118]
Anodized epitaxial graphene (EG)	Defects generated on anodized EG surface	Decreased by anodization	Increased by anodization	N/A	N/A	81	0.00981 in [Ru(NH_3_)_6_]^3+/2+^ 0.00101 in [Fe(CN)_6_]^3−/4−^	5.55 at 0.25 V	[58]
CRGO/GCE	Curly with 100 nm thickness and 0.8 nm single sheet	0.117	1.38	160.8	0.092	N/A	N/A	N/A	[106]

## Aspects affecting stability and/or reproducibility of electrochemical biosensors

**Fig. 5:**
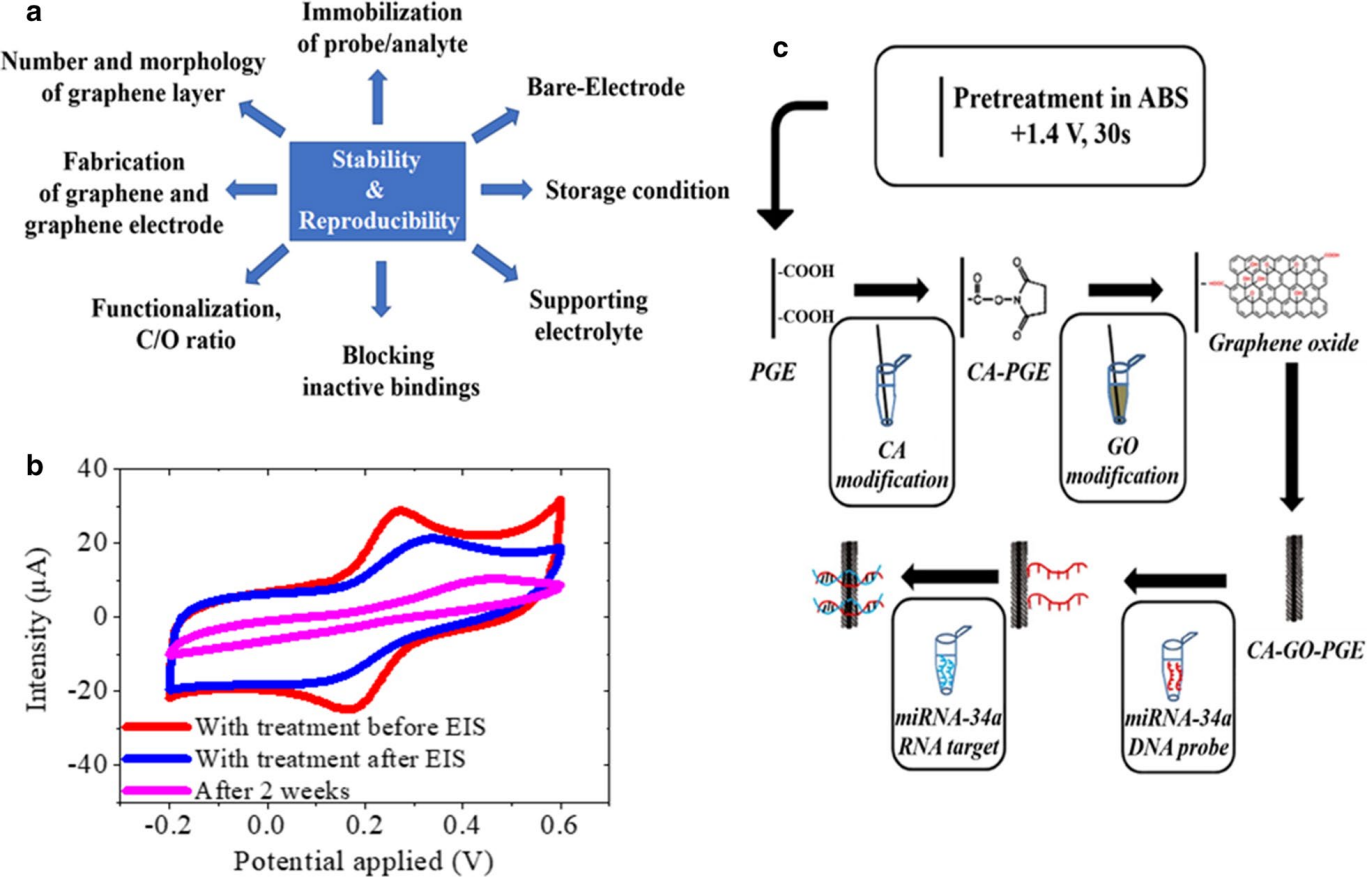
Reproducibility and stability of graphene electrodes for biosensing. **a** Factors that influence the stability and reproducibility of graphene biosensors. **b** CV curves of reduced graphene oxide by two-step approach (i.e. reduction after drop-casting) after different acquisition time obtained in 0.1 M KCl solution containing 1 mM K_3_[Fe(CN)_6_]/K_4_[Fe(CN)_6_]. **c** The representative scheme of experimental procedure followed in the hybridization occured between miRNA-34a target and its complementary DNA probe at the surface of CA/GO/PGEs (Reprinted with permission from [86]; copyright 2017 Elsevier)

Graphene’s discovery in 2004 introduced unique properties revolutionizing preconceptions that such 2D materials were thermodynamically unstable. However, reliable stability and biosensor reproducibility remain a major problem for consistent performance within large-scale technological applications. Electrochemical sensor device reliability is influenced by limitations concerning electrode pretreatments, precise synthesis of graphenic materials [119] and device architectures. The sensing performance may differ from device-to-device even though the graphene materials originate from the same batch and fabrication protocol. Key factors that could influence the stability and reproducibility, taking graphene as a benchmark material are portrayed in Fig. 5a.

Conversely, GO is a metastable material that may undergo spontaneous reduction under certain conditions such as exposure to sunlight [11] and its stability and aggregation have been investigated in various media. In aqueous solution, ionic strength, pH, ion valence and presence of organic matter all significantly influence GO properties in solution. Its stability is also strongly influenced by the interdependent factors of surface oxidation and polarity. Regarding polarity, a GO sheet is amphiphilic, bearing hydrophilic sites (–COOH at the edge plus phenol and hydroxyl groups at the basal plane) and graphitic domain hydrophobic sites at the basal plane. Furthermore, the colloidal stability of GO sheets depends on their edge-to-edge interacting geometry. Electrostatic repulsion tends to dominate, but if brought face-to-face, the attractive Van der Waals and π–conjugated interactions may predominate [120]. This helps explain the aggregative behavior of GO during storage and simple GO redispersion by sonication. Disaggregated into small flakes, GO sheets tend to restack together [70]. The stability and reproducibility of graphene electrodes, are strongly influenced by the concentration of GO sheets and extent of dispersion in solution. Good dispersion is thus already a critical parameter to take into account, as are the polarity of GO sheets and pH of the solution. Cote et al. showed by Zeta potential measurements that the hydrophilicity of GO increased with pH and decreasing the sheet size from a micron to a nanometer scale (< 100 nm) lowering the extent of reduction [120].

The stability of graphene oxide in water is affected by subsequent reduction of its functional groups, and by pH which is known to have the greatest influence on rGO stability [11]. Figure 5b illustrates a significative change of rGO CV signal over time. Nafion and lysozyme are good dispersants for rGO [121, 122]. Functionalization could increase the performance of carbon-based materials. Graphene based composites with polymers or inorganic compounds may offer more sensitivity, selectivity and reproducibility. The combination of 2D materials with appropriate interface materials represents a successful approach to improve the sensitivity and stability of a biosensing platform [123]. Important binding agent members such as chitosan, dopamine or nafion can increase graphenic stability and fix molecules tightly onto the electrode surface [2, 5, 44], thereby enhancing the adherence of GO onto the electrode surface. Composites made by mixing graphene with metallic nanoparticles may open new doors towards highly stable and reproducible biosensors. In a complementary manner, chemical activation procedures may help stabilize the surfaces of electrodes and provide better attachment for graphene onto surface [124] (Fig. 5c). When not used, it is generally advised that graphene electrodes be kept at 4 °C for stability [106], as recommended for other 2D materials [18]. Electrochemical measurements based on field-effect (FET) technology encouraged interest in 2D materials for sensors due to their sensitivity, fast response and real-time monitoring. New composite formulations can resolve problems such as the fragility of black phosphorus, readily degraded by water vapor, oxygen and irradiation from visible light [123]. By doping BP with sulphur, the innovative FET device still retained useful conductivity after exposure to air for 21 days with a decrease of charge-carrier mobility of only 22.6% [125].

With respect to device architecture, clear electrochemical differences are introduced by the choice of aptamer binding method, either by simple adsorption (Table 3) or involving covalent interaction (Table 4). Regarding DNA probe immobilization at the surface of the WE by simple adsorption, hexagonal cells of graphene sheets and the aromatic rings of nucleobases share Van der Waals forces, ionic interactions, π–π stacking, and hydrogen bonds. DNA is physically adsorbed better on reduced GO than GO, since rGO has more aromatic regions for π–π stacking interactions with DNA bases and a lower surface charge density lessens electrostatic repulsion of the negatively charge DNA backbone [87, 107, 126].

**Table 3:** Biosensors with nucleic acid aptamer immobilization using noncovalent approach

Electrode	DNA aptamer probe [concentration]	Immobilization (buffer)	Wash and drying conditions	Analyte target [concentration]	Aptamer-analyte hybridization (buffer)	Washing and drying	Electrolyte	Stability	Reproducibility	References
DEP-GO; DEP-ERGO	THR-APT: 5′-TTT TTT TTT TTT TTT GGT TGG TGT GGT TGG-3′ [10 µM]	3 μL of THR-APT deposited onto modified electrode surface for 10 min at 60 °C in (PBS)	Wash in PBS gentle stirring Dry at room temperature	THR [40.5 nM]	Incubated in 100 µL with THR for 60 min. at 37 °C in (Tris buffer)	Tris buffer solution at 37 °C 2×	EIS in 10 mM K_3_[Fe(CN)_6_]/K_4_[Fe(CN)_6_] in TBS (pH 8.2)	N/A	N/A	[107]
Graphene modified DEP chip	hpDNA [10 µM]	3 μL hpDNA probe drop onto modified electrode surface for 10 min at 60 °C in (TSC1)	Wash in TSC1, gentle stirring Dry at room temperature	cDNA target [30 nM]	Incubated in 100 µL with DNA target for 30 min at 55 °C in (TSC1)	TSC2 buffer at 42 °C 2×	EIS in 0.1 M PBS containing 10 mM K_3_[Fe(CN)_6_]/K_4_[Fe(CN)_6_]	N/A	Low for G-SL RSD = 31%	[127]
rGO-graphene double-layer electrode	Fluorescent dye labelled HIV1 gene aptamer 5ʹ-AGT CAG TGT GGA AAA TCT CTA GC-carboxyfluorescein-3ʹ [1 µM]	10 µl ssDNA probe deposited onto modified electrode surface at 35 °C for 30 min in (PBS)	Wash in nuclease free water 15×	5ʹ-GCT AGA GAT TTT CCA CAC TGA CT-3ʹ [0.01–100 nM]	Dripped 10 µL target cDNA onto electrode surface at room temperature for 1 h in (PBS)	Nuclease free water	DPV, CV in 10 mM ferricyanide aqueous solution (1 M KCl as support electrolyte)	~ 3 h	RSD = 6.2–8.1%	[135]
ERGO (DEP chip)	hpDNA: 5′-ATG GAG ACC AGG CGG CCG CAC ACG TCC TCC AT-3′ [10 µM]	hpDNA probe dry-adsorbed onto modified electrode surface at 60 °C for 10 min in (PBS)	Wash in PBS 1× Dry at room temperature	ssDNA target: 5′-ATG GAG GAC GTG TGC GGC CGC CTG GT-3′ [0.01–10 nM; 300 nM]	Incubated in 100 µL with ssDNA target for 40 min at 55 °C in (SSC)	SSC buffer at 42 °C 2×	EIS in 10 mM of [Fe(CN)_6_]^3−/4−^ as redox probe in 0.1 M PBS solution.	N/A	N/A	[108]
Graphene microarray grown by CVD	5′-AGC TTC ATA ACC GGC GAA AGG CTG AAG CT-3′ [10 µM]	10 μL DNA probe adsorbed on graphene surface for 2 h in (10 mM PBS/150 mM NaCl/50 mM MgCl_2_)	Wash in buffer	5′-AGC TTC AGC CTT TCG CCG GTT ATG A-3′ [5 pM to 50 nM]	Incubation in DNA target solution N/A	N/A	CV and EIS in 1 mM K_3_Fe(CN)_6_, 1 mM K_4_Fe(CN)_6_ and 10 mM PBS	N/A	N/A	[126]

**Table 4:** Biosensors with nucleic acid aptamer immobilization covalent approach

Electrode	Electrode surface Activation	DNA aptamer probe (size) sequence [concentration]	Electrode surface Immobilization	Analyte target [concentration]	Aptamer-Analyte Nucleic acid Hybridization	Electrolyte	Stability	Reproducibility	References
DEP chips GCE	0.05 M EDC (3 µL) and 0.03 M sulfo-NHS in PBS (pH 7) for 1 h to activate carboxyl acid groups. PBS wash	DNA (26 base) 5′-NH_2_-(CH_2_)_6_-ACCAGGCGGCCGCACACGTCCTCCAT-3′ [N/A]	3 µL DNA probe at optimized concentration deposited overnight in humidified air at RT in PBS (pH 7)	5′-ATG-GAGGACGTGTGCGGCCGCCTGGT-3′ N/A	Incubated with gentle stirring in 100 µL with DNA target for 30 min at 42 °C in (TSC1 buffer)	CV and EIS in 0.01 M PBS buffer containing K_3_[Fe(CN)_6_]/K_4_[Fe(CN)_6_] (10 mM) 1:1 molar ratio	N/A	N/A	[136]
GCE/rGO/AMEL	0.2 M EDC in MES buffer and 0.5 M NHS in 0.1 M PBS treatment for 1 h to activate carboxyl acid groups of rGO. PBS wash	AMGX (17 base) 3′-TATCCC AGATGT TTCTC-NH_2_-5′ [1 µM]	80 µL DNA probe dispensed on inverted surface covered with a glassy cap of for 1 h at 25 °C	AMGX: 3′-GAGAAACATCTGGGATA-5′ [10 zM–10 fM]	With PCR	0.5 mM [Fe(CN)_6_]^3−/4−^ in 0.1 M KCl	3 days	RSD = 5.014%	[48]
Anodized epitaxial graphene electrode	0.2 M EDC and 0.5 M NHS for 1 h. Rinsed with ultra-pure water	DNA (30 base) NH_2_-C_12_-5′-GCACCTGACTCCTTGGAGAAGTCTGCCGT-3′ [N/A]	DNA solution added and incubated overnight	5′-ACG GCA GAC TTC TCC ACA GGA GTC AGG TGC-3′ [50 fM–1 µM]	Electrode treated with 100 μL ssDNA target solution for 40 min at 42 °C	EIS in PBS/14 mM NaCl/0.27 mM KCl/1 mM Na_3_PO_4_ and 0.176 mM K_3_PO_4_)	N/A	7% for 1 nM	[137]
MnTPP/rGO-GCE	20 mM EDC and 32 mM NHS in 10 mM PBS for 1 h at room temperature	ssDNA (25 base) NH_2_-C_6_-5′-TCAATCTCG GGAATCTCA ATGTTAG-3′ [1 µM]	Add a solution of ssDNA probe for 1 h at room temperature in the activation solution	5′-CTAACATTG AGATTCCCGAGATTGA-3′ [100 aM–1 nM]	5 μL DNA target deposited for 40 min at 47 °C.	0.1 M KCl with 5 mM [Fe(CN)_6_]^3−^/[Fe(CN)_6_]^4−^	High	RSD < 3%	[138]
Gold-wire electrode (AuE)	N/A	Thiol-ssDNA (23 base) 5′-SH-(CH_2_)_6_-AGTCAGTGT GGAAAATCT CTAGC-3′ [N/A]	2 µL of ssDNA dropped on AuE surface and kept 15 min in water at room temperature. Then immersed in dispersed graphene for 15 min	DA [1–50 nM]	N/A	DPV in 0.2 M of PBS buffer (pH 7.4)	1 week; 90% in 2 weeks	3.5% for 1 µM DA	[139]
MoS_2_/graphene/GCE	N/A	DNA (15 base) 5′-AGTGATTTT AGAGAG-3′ [1 µM]	20 µL pDNA dropcast on MoS_2_/graphene/GCE. Dried in oven for 35 min at 57 °C	5′-TCA CTA AAA TCT CTC-3′ [0.1–100 fM]	20 µL cDNA dropcast on GCE, dried for 30 min at 57 °C. Treated with 1 M KCl solution 0.2 M K_3_[Fe(CN)_6_] at - 0.7 V for 300 s to release the dsDNA.	1.0 M KCl solution containing 0.2 M K_3_ [Fe(CN)_6_] at a scan rate of 0.10 V/s from 0.6 to − 0.3 V	N/A	N/A	[140]
AuNP/graphene–VS_2_/GCE	N/A	DNA (S1) (21 base) 5′-HS-(CH_2_)_6_-TTGCCCGTTTACTTTGGGTCT-3′ [9.6 µM]	AuNPs/grapheneVS2/GCE was incubated in HS-DNA for 3 h at room temperature	5′-AGA CCC AAA GTA AAC GGG CAA-3′ [0.5–500 pM]	Immersion of the electrode into target DNA at room temperature for 60 min	1 mmol/L [Fe(CN)_6_]^3−/4−^ containing 0.1 mol/L KCl	92.1% after 1 week	RSD = 4.3%	[141]
GO-PGE	N/A	HBV DNA probe: (20 base) 5′-NH_2_-(CH_2_)_6_-AATACCACA TCATCCATA TA-3′ [40 µg/mL]	GO-PGEs were immersed in 110 µL of amino linked HBV probe solution prepared in ABS for 1 h	5′-TAT ATG GAT GAT GTG GTA TT-3′ [160 µg/mL]	Probe immobilized electrodes immersed in 110 µL target for an hour	EIS in 2.5 mmol/L K_3_[Fe(CN)_6_]/K_4_[Fe(CN)_6_] (1:1) with 0.1 mol/L KCl DPV in ABS	N/A	RSD = 14.2%	[142]
GQD-PGE	N/A	ssDNA-1 (35 base): 5′-TCTCTCAGT CCGTGGTAG GGCAGGTTG GGGTGACT-3′ [500 nM]	Electrode immersed in 70 μL 10 mM Tris–HCl buffer containing ssDNA-1 for 1 h	5′-AG TCA CCC CAA CCT GCC CTA CCA CGG ACT GAG AGA-3′ [100–500 nM]	Target first added to solution of ssDNA-1 and incubated for 1 h before the immobilization	DPV in 10 mM tris–HCl buffer solution containing 5 mM [Fe(CN)_6_]^3−/4−^ pH 6	N/A	N/A	[143]
GCE-APTES-rGO	N/A	MRSA DNA probe (30 base) 5′-ATGATTATG GCTCAGGTA CTGCTATCC ACC-3′ [10 µM]	5 μL DNA dropped onto GCE-APTES-rGO electrode then capped with a centrifuge tube and kept at room temperature for 6 h	5′-GGT GGA TAG CAG TAC CTG AGC CAT AAT CAT-3′ [10 µM]	5 μL target DNA solution dropped onto GCE’s and droplet kept at room temperature for 30 min	0.1 M KCl containing 5 mM [Fe(CN)_6_]^3−/4−^ (1:1)	N/A	N/A	[144]
PTCA/CCG-GCE	200 mM EDC and 50 mM NHS in MES buffer cast on PTCA/ CCG-GCE surface to activate the carboxyl group for 1 h. Rinsed with 10 mm Tris buffer (pH 7.4)	AS1411 (32 base) 5′-GGTGGT GGTGGTTGT GGTGGTGGT GGTTTTTT-NH2-3′ [1 µM]	DNA dropcast on the surface and then incubated for 4 h	Cancer cells [1 µM]	The surface was washed by buffer and subsequently hybridized with aptamer DNA in 10 mM Tris, 2.5 mM MgCl_2_, 140 mM KCl (pH 7.4) for 1 h	CV in 10 mM K_3_[Fe(CN)_6_], 1.0 M KCl; EIS in 10 mM K_3_[Fe(CN)_6_]/K_4_[Fe(CN)_6_] (1:1) mixture with 1.0 M KCl	N/A	N/A	[145]

Notably, when using [Fe(CN)6]^3−/4−^ as the redox probe, an increased impedimetric response upon physical adsorption of the DNA probe preceded a decreased impedance signal after hybridization with the target. Inferred partial release of the dsDNA from the electrode could decrease the total charge present on the electrode surface, thus reducing the charge transfer resistance [127]. Nonetheless simple adsorption techniques can be advantageously mild and Raman analysis indicated no introduced defects or disorder on the graphene structure [128].

In contrast, covalent bonding immobilizes the DNA probes at one end, improving stability with protection from desorption. This approach can achieve good vertical orientation of the biomolecules on the electrode surface, favouring efficient aptamer/analyte hybridization. Unlike adsorption-based methods, the charge transfer resistance continued to increase upon analyte hybridization due to a more negatively charged species on the surface of the electrode and repulsion of [Fe(CN)6]^3−/4−^ by the negatively charged phosphate backbone of the hybrdized DNA probe [129]. This improved sensitivity for electrochemical analyte detection.

## Conclusions

Ubiquitous development of electrochemical biosensors for healthcare and biomedicine has yet to produce fully integrated functional devices approved for real clinical evaluation. Nonetheless, the growing portfolio of biosensors using unique versatile 2D nanomaterials and their hybrid composites as a thin surface veil imparting transformative properties has encouraging potential for the biomedical field. Manufacturing graphene sheets to perfection need not be essential, so long as measurement conditions and quality control can maintain a satisfactory consistency and reproducibility. Although defects are often considered detrimental to material properties, if engineered to tailor the surface in a controlled manner, they can introduce properties that enhance function for a wide range of new electrochemical biosensors.

Research into innovative 2D material-based sensors beyond the first application of graphene in 2008, has provided useful experimental examples, yet for commercialization the technology is still in its infancy, facing challenging fabrication procedures and poor control of uniformity and reproducibility for reaching the desired properties [130]. There is still need for much research into fundamental aspects of this field. Reports of biosensors demonstrating relatively good stability and reproducibility often combine graphene with composite compounds [131], modulate graphene via doping [132], introduce covalent approaches for target detection and/or place emphasis on carefully controlled storage conditions. It is anticipated that progress in understanding the mechanism for the excellent electro-catalytic activity of 2D materials will improve prospects for meeting the urgent need for point of care (POC) devices [133] and live cell monitoring [134], through low-cost miniaturized potentiostats. Biosensor nanotechnology provides one of the most pertinent examples where success depends upon multidisciplinary collaboration supported by partnership between public research centres, industry and the health sector.

## Data Availability

Not applicable.

## Abbreviations

0D: zero-dimensional
1D: one-dimensional
2D: two-dimensional
3D: three-dimensional
AA: ascorbic acid
ABS: acetate buffer solution
AC: alternating current
a-C: amorphous carbon
aM: attomolar
AMEL: amelogenin
AMGX: proprietary name for AMEL complementary probe sequence
APBA: 3-aminophenylboronic acid
Apt: aptamer
APTES: 3-aminopropyltriethoxysilane
AuE: gold-wire electrode
AuNPs: gold nanoparticles
BP: black phosphorus
CA: chronoamperometry
CB: carbon black
CCG: chemical converted graphene
cDNA: complementary DNA
CE: counter electrode
cm: centimeter
CNTs: carbon nanotubes
CR: chemical reduction
CRGO: chemically reduced graphene oxide
CV: cyclic voltammetry
CVD: chemical vapor deposition
DA: dopamine
DC: direct current
DEP-chip: disposable electrical printed chip
DNA: deoxyribonucleic acid
DOS: density of state
DPV: differential pulse voltammetry
dsDNA: double-stranded DNA
E: electrode potential
EDC: 1-ethyl-3(3-dimethylaminopropyl)-carbodiimide
EG: epitaxial graphene
EIS: electrochemical impedance spectroscopy
ER: electrochemical reduction
ERGO: electrochemically reduced graphene oxide
ET: estriol
[Fe(CN)_6_]^3−/4−^: ferrocyanide/ferricyanide ions
FET: field-effect transistor
fM: femtomolar
GCE: glassy carbon electrode
GO: graphene oxide
GOx: glucose oxidase
GQDs: graphene quantum dots
Gr-HPHT: graphene-high pressure, high temperature
G-SL: single-layer graphene
HBV: hepatitis B virus
HET: heterogeneous electron transfer
HIV 1: human immunodeficiency virus 1
hpDNA: hairpin DNA
HRGO: hydrothermally reduced graphene oxide
Hz: Hertz
I: Current
I_D_/I_G_ ratio: ratio of the intensity of D and G Raman peaks
K_3_[Fe(CN)_6_]: potassium ferricyanide
K_3_PO_4_: tripotassium phosphate
K_4_[Fe(CN)_6_]: potassium ferrocyanide
KCl: potassium chloride
k^o^: heterogenous electron transfer rate constant
Ks: electrode transfer rate
L_a_: lateral crystallite size
LOD: limit of detection
M: molar
MES: 2-(*N*-morpholino)ethanesulfonic acid
mg: milligram
MgCl_2_: magnesium chloride
miRNA: microRNA
mL: milliliter
ML: multilayer
mM: millimolar
MnTPP: manganese (III) tetraphenylporphyrin
MoS_2_: molybdenum disulfide
MRSA: methicillin-resistant *Staphylococcus aureus*
mV: millivolts
mV/s: millivolts per second
MWCNTs: multi-wall carbon nanotubes
N/A: not applicable/available
N_2_: nitrogen
Na_2_SO_4_: sodium sulfate
Na_3_PO_4_: trisodium phosphate
NaCl: sodium chloride
NADH: nicotinamide adenine dinucleotide
NaOH: sodium hydroxide
nc-G: nanocrystalline graphite
NHS: *N*-hydroxysulfosuccinimide
NL: number of layers
nm: nanometer
nM: nanomolar
PBS: phosphate buffer solution
PCR: polymerase chain reaction
pDNA: probe DNA
pg: picogram
PGE: pencil graphite electrode
pM: picomolar
POC: point of care
PTCA: 3,4,9,10-perylene tetra-carboxylic acid
PVD: physical vapor deposition
QDs: quantum dots
Rct: charge transfer resistance
RE: reference electrode
rGO: reduced graphene oxide
RNA: ribonucleic acid
RSD: relative standard deviation
RT: room temperature
[Ru(NH_3_)_6_]^3+/2+^: hexaammineruthenium (III)/(II)
s: second
S/N: signal-to-noise ratio
SBS: salbutamol sulfate
SiC: silicon carbide
SNPs: single-nucleotide polymorphisms
SPCE: screen printed carbon electrode
SPE: screen printed electrode
SSC: saline sodium citrate
ssDNA: single-stranded DNA
SWV: square wave voltammetry
t: time
ta-C: tetrahedral amorphous carbon
TBS: tris–HCl buffered saline
Thi: thionine
THR: thrombin
TMDs: transition metal dichalcogenides
TRGO: thermally reduced graphene oxide
TSC: trisodium citrate
UA: uric acid
V: volts
VS_2_: vanadium disulfide
WE: working electrode
zM: zeptomolar
µA: microampere
µF: microfarad
µg: microgram
µL: microliter
µM: micromolar
ϕ: phase
ω: frequency
Ω: ohm
